# Identification of Two Homozygous Variants in *MYBPC3* and *SMYD1* Genes Associated with Severe Infantile Cardiomyopathy

**DOI:** 10.3390/genes14030659

**Published:** 2023-03-06

**Authors:** Marta W. Szulik, Miguel Reyes-Múgica, Daniel F. Marker, Ana M. Gomez, Matthew D. Zinn, Leslie K. Walsh, Juan Pablo Ochoa, Sarah Franklin, Lina Ghaloul-Gonzalez

**Affiliations:** 1 Nora Eccles Harrison Cardiovascular Research & Training Institute, University of Utah, Salt Lake City, UT 84112, USA; 2 Division of Pediatric Pathology, Department of Pathology, University of Pittsburgh, Pittsburgh, PA 15224, USA; 3 Division of Neuropathology, Department of Pathology, University of Pittsburgh, Pittsburgh, PA 15213, USA; 4 Division of Cardiology, Department of Pediatrics, University of Pittsburgh, Pittsburgh, PA 15224, USA; 5 Division of Genetic and Genomic Medicine, Department of Pediatrics, University of Pittsburgh, Pittsburgh, PA 15224, USA; 6 Biomedical Research Institute of A Coruña, 15006 A Coruña, Spain; 7 Cardiovascular Genetics, Health In Code, 15008 A Coruña, Spain; 8 Division of Cardiovascular Medicine, Department of Internal Medicine, University of Utah School of Medicine, Salt Lake City, UT 84112, USA; 9 Department of Human Genetics, University of Pittsburgh, Pittsburgh, PA 15224, USA

**Keywords:** infantile cardiomyopathy, MYBPC3, SMYD1, biventricular heart failure, hypertrophic cardiomyopathy

## Abstract

Mutations in cardiac genes are one of the primary causes of infantile cardiomyopathy. In this study, we report the genetic findings of two siblings carrying variations in the *MYBPC3* and *SMYD1* genes. The first patient is a female proband exhibiting hypertrophic cardiomyopathy (HCM) and biventricular heart failure carrying a truncating homozygous *MYBPC3* variant c.1224-52G>A (IVS13-52G>A) and a novel homozygous variant (c.302A>G; p.Asn101Ser) in the *SMYD1* gene. The second patient, the proband’s sibling, is a male infant diagnosed with hypertrophic cardiomyopathy and carries the same homozygous *MYBPC3* variant. While this specific *MYBPC3* variant (c.1224-52G>A, IVS13-52G>A) has been previously reported to be associated with adult-onset hypertrophic cardiomyopathy, this is the first report linking it to infantile cardiomyopathy. In addition, this work describes, for the first time, a novel *SMYD1* variant (c.302A>G; p.Asn101Ser) that has never been reported. We performed a histopathological evaluation of tissues collected from both probands and show that these variants lead to myofibrillar disarray, reduced and irregular mitochondrial cristae and cardiac fibrosis. Together, these results provide critical insight into the molecular functionality of these genes in human cardiac physiology.

## 1. Introduction

Infantile cardiomyopathies affect at least one per 100,000 children < 18 years old in the USA alone and have severe impacts on pediatric morbidity and mortality [1]. Around 40% of symptomatic children either undergo heart transplant or die within 2 years [2]. In this population, genetic mutations are becoming increasingly more recognized as causative. While hypertrophic cardiomyopathy (HCM) is the most commonly inherited cardiac disorder [1,3], mutations in sarcomeric genes are currently the most commonly known cause of cardiomyopathy in children [1,2]. In addition, HCM can not only result from significant genetic heterogeneity but can also produce substantial phenotypic heterogeneity between individuals with mutations in the same gene or even with the same mutation [1]. While genetic testing and evaluation has improved the ability to identify genetic variants underlying the etiology, it is not always routinely and universally incorporated into the clinical care of children with cardiomyopathy. Therefore, it is thought that many pathogenic variants in pediatric patients remain unidentified.

MYBPC (myosin-binding protein-C) is a modular polypeptide protein that functions as a structural component of the striated muscle sarcomere. There are three isoforms of MYBPC in humans: MYBPC1 is expressed in slow skeletal muscle, MYBPC2 is expressed in fast skeletal muscle and MYBPC3 is only expressed in cardiac tissue [4,5]. MYBPC3 is a key component of the thick filaments, where it binds to myosin, titin and actin and functions to maintain structural integrity of the sarcomere and regulate cardiac contractility [6,7,8,9,10]. Currently, there are >350 individual variants that have been identified in the *MYBPC3* gene; most of these are truncating mutations (>60%) affecting adults [11]. The vast majority of *MYBPC3*-associated HCM mutations are heterozygous, and patients often have disorganized sarcomere structures in the heart and manifest, as late adult-onset symptoms, with a slow disease progression [10,12,13]. In the pediatric population, it has been reported that 14% of pediatric hypertrophic cardiomyopathy cases are caused by compound heterozygous variants in *MYBPC3,* which occur when both copies of a particular gene each carry a mutation (not necessarily the same mutation) [10].

Intronic variants in *MYBPC3* in the context of hypertrophic cardiomyopathy have also been identified [14,15,16] and, in the last few years, have focused on one specific intronic *MYBPC3* variant c.1224-52G>A (IVS13-52G>A) [17,18,19,20,21]. Specifically, Bagnall et al. reported on a cohort of unrelated gene-elusive probands with HCM that had undergone genome sequencing, in which they identified the intronic *MYBPC3* variant c.1224-52G>A in two adult male family members, as well as an adult proband from another family [17]. In a separate study by Harper et al., this *MYBPC3* c.1224-52 G>A variant was shown to be responsible for the HCM phenotype previously attributed to the c.3628-41_3628-17del polymorphism in the South Asian population [18]. In another study, led by Lopes et al., they performed large-scale unbiased screening of intronic variants in *MYBPC3* in 1644 unrelated and consecutive patients with HCM and identified 18 adults carrying the *MYBPC3* c.1224-52 G>A variant [20]. Interestingly, co-segregation of this variant with HCM was demonstrated in three families [20]. Finally, in the most recent study published by Torrado et al., they analyzed a large HCM cohort in which they identified 12 adult carriers of this specific *MYBPC3* c.1224-52G>A variant [21]. Thus, to date, this variant has been identified independently and in conjunction with other variants in adult patients with hypertrophic cardiomyopathy [17,18,19,20,21,22]. 

SMYD1 (SET and MYND domain-containing protein 1) is a lysine methyltransferase, which has been shown to methylate lysine 4 on histone H3, an established mark of gene activation [23,24,25,26,27]. SMYD1 is only expressed in skeletal and cardiac muscle [28,29,30] and was shown to play a significant role in regulating cardiac development [29]. Specifically, the constitutive loss of *Smyd1* in mice in utero leads to death at embryonic stage E9.5 due to cardiac defects, manifested by the disrupted maturation of ventricular cardiomyocytes, malformation of the right ventricle and truncation of the outflow tract [29,31]. It has also been shown that the conditional cardiomyocyte-specific loss of *Smyd1* is lethal at embryonic stage E12.5–15.5, accompanied by pericardial edema, thinned pericardium and decreased trabeculation [31]. In the adult myocardium, the loss of *Smyd1* using inducible, cardiomyocyte-specific *Smyd1* knockout mice leads to the massive downregulation of mitochondrial bioenergetics [27], cardiac hypertrophy, fibrosis and heart failure [32]. While SMYD1 has been the subject of several studies in zebrafish and mice, since it was first identified, only in the last few years have human patients been identified with variants in the *SMYD1* gene exhibiting cardiomyopathies. There are currently three previous reports of patients with *SMYD1* variant-associated cardiomyopathies: the first was a 24-year-old patient with hypertrophic cardiomyopathy and a de novo heterozygous variant in the *SMYD1* gene (c.814T>C; p.Phe272Leu) [33]; the second patient displayed left ventricular noncompaction (LVNC) cardiomyopathy and arrhythmias who underwent heart transplantation and has a truncating heterozygous variant in the *SMYD1* gene (c.675delA; p.Lys225Asnfs*8) [34]; the third report described a family where two siblings, male (7 days old) and female (4 years old), were both diagnosed with congenital heart disease (aortic valve stenosis “AVS” and bicuspid aortic valve “BAV”) and tested positive for a heterozygous variant in the *SMYD1* gene (c.1321C>T; p.Arg441Trp), in addition to a *BMP10* variant (c.625C>T; p.Arg209Cys) [35].

Here, we report a patient with biventricular heart failure carrying two homozygous variants. The first variant identified was a homozygous variant (c.302A>G; p.Asn101Ser) in the *SMYD1* gene via whole exome sequencing. This variant has never been reported before, although this patient was briefly mentioned in a previously published case report, which described the patient’s original presentation and medical care during the biventricular assist device (BiVAD) implant and subsequent cardiac transplant. Even though the report mentioned the detection of a *SMYD1* variant, no details about the variant were reported [36]. Since the original report, we have followed the patient clinically and consented the patient, her parents and siblings to a research study for further functional analyses. In this report, we present the whole exome sequencing (WES) results of this proband (P1), which revealed the presence of a novel homozygous missense *SMYD1* variant (c.302A>G; p.Asn101Ser), as well as a homozygous truncating *MYBPC3* variant (c.1224-52G>A, IVS13-52G>A), the latter variant detected in her younger male sibling (P2), who was also diagnosed with infantile cardiomyopathy. Familial testing for these two variants was performed for the rest of the family. We also performed a histopathological analysis of the skeletal and cardiac tissues from the probands, which show cellular abnormalities in myofibers and mitochondrial structures, including the analysis of cristae. These results constitute the first analysis of the *MYBPC3* (c.1224-52G>A, IVS13-52G>A) and *SMYD1* (c.302A>G; p.Asn101Ser) variants in human tissue, functionally link genetic variations to cardiomyocyte dysfunction and provide key biological insights into the development of cardiomyopathies in pediatric patients.

## 2. Materials and Methods

### 2.1. Human Studies

Informed consent was obtained and approved for all participants in accordance with the University of Pittsburgh IRB protocol #19040093. For underaged participants, parental informed consent was obtained and approved. All methods were performed in accordance with the relevant guidelines and regulations outlined by the IRB.

### 2.2. Whole Exome Sequencing/Familial Genetic Testing

Whole exome sequencing (WES) was performed on a clinical basis on the affected female proband (P1) and both unaffected parents by GeneDx (GeneDx, Gaithersburg, MD, USA). The raw sequencing data was not provided from the clinical lab; only variants identified from the sequencing were released. Targeted genetic testing for the *MYBPC3* c.1224-52G>A (IVS13-52G>A) and *SMYD1* (c.302A>G; p.Asn101Ser) variants was performed on the affected male proband (P2) and two unaffected brothers.

### 2.3. Histology and Ultrastructural Analysis

Muscle from the right thigh (from P1) and tissue from the left and right ventricles of the P1 and P2 were obtained. The skeletal muscle biopsy was performed during the clinical workup of the P1 patient at three months of age. For both P1 and P2 patients, cardiac tissue was obtained from the entire heart after transplant. For light microscopy sections, tissues were immediately fixed in 10% formalin, embedded in paraffin, and histologic sections cut at 4 µm, mounted on charged slides and stained using well-established methods for hematoxylin and eosin (H&E). Photomicrographs were obtained using an Olympus microscope or capturing scanned digital images. Skeletal muscle tissue was also evaluated using Gomori’s trichrome stain, according to the manufacturer’s instructions. For the evaluation of fibrosis, cardiac tissue was stained with Masson’s trichrome according to the manufacturer’s instructions. Immunohistochemistry for Desmin was performed with prediluted clone DE-R-11(R) from Roche Tissue Diagnostics (Indianapolis, IN, USA). For the ultrastructural analysis, muscle and heart samples were fixed in Karnovsky’s solution (2% Paraformaldehyde/2.5% Glutaraldehyde in Sodium Cacodylate Buffer), followed by Epon embedding, ultramicrotomy sectioning and staining with uranyl acetate and lead citrate. Ultrathin sections (50 to 100 nm) were prepared and examined with a Phillips transmission electron microscope.

## 3. Results

### 3.1. Clinical Description of the Female Proband (P1)

**Figure 1:**
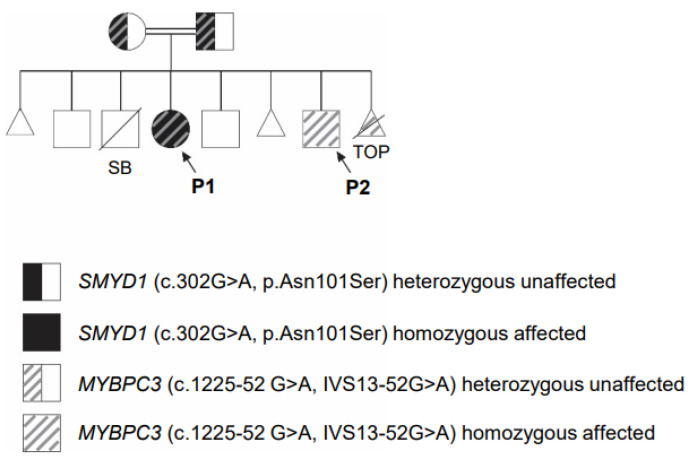
Pedigree of the family carrying the *SMYD1* (c.302A>G; p.Asn101Ser) variant and *MYBPC3* c.1224-52 G>A (IVS 13-52G>A) variant. Females are represented by circles, males are represented by squares, miscarriages are represented by triangles, termination of pregnancy (TOP) is represented by a triangle and diagonal line, diagonal line indicates deceased and double horizontal lines indicate consanguinity. Both parents are unaffected and heterozygous for both variants, while P1 is homozygous for both *SMYD1* and *MYBPC3* variants and P2 is homozygous for the *MYBPC3* variant (arrows). TOP is homozygous for *MYBPC3*. The two unaffected male children are not carriers for either gene variant. Male stillborn (SB) deceased at 7 months gestation was not tested.

A brief report of the proband P1 carrying the novel *SMYD1* variant was published in 2019 [36], and some of the details have also been included here for a comprehensive overview of the phenotype, including the proband’s original presentation, medical care requiring biventricular assist device (BiVAD) implant and subsequent cardiac transplant. P1 was a female child of unaffected, healthy parents in a consanguineous relationship (first cousins). The proband’s parents have ethnic backgrounds from the Middle East (mother: Qatar and Syria, father: Qatar). The child was delivered at full term via scheduled C-section following an uncomplicated pregnancy to a mother who had previously experienced one early first trimester miscarriage, one healthy pregnancy that resulted in a healthy male child and a third pregnancy that resulted in a stillborn male child (secondary to nuchal cord) with the same partner (Figure 1). The proband’s older male sibling was unaffected and presented with a normal echocardiogram. At birth, P1 appeared normal until the third day after birth, when she developed systemic desaturation responsive to supplemental oxygen. The initial echocardiogram showed normal biventricular function. Over the next 2 weeks, her left ventricular ejection fraction (LVEF) fell to 38% but improved after the initiation of milrinone and eventual transition to oral heart failure therapy. She also developed ectopic atrial tachycardia that was responsive to amiodarone. Cardiac catheterization showed normal coronary artery anatomy and antegrade blood flow. Despite treatment and initial stabilization, the patient deteriorated rapidly over the next two months, as manifested by progressive biventricular heart failure (ejection fraction of <30%) and recurrent tachyarrhythmia. The patient received biventricular mechanical circulatory support as a successful bridge to cardiac transplantation at four months of age [36].

In this report, we have included updated information on the patient’s health and well-being since the original description. P1 was seen in the medical genetics clinic post-heart transplant at age 2 years and 11 months old, as well as 5 years of age. She did not have any signs or symptoms of muscle hypotonia during the exam, and no developmental concerns were noted or reported during both clinical examinations, even though the patient received physical therapy previously due to prolonged hospitalization for heart failure/heart transplant and recovery. The patient had her first steps at 15 months of age after using a walker for several months, and at the day of her genetics follow-up at ~3 years of age, her parents reported that she was able to run and climb up and down the stairs with no issues. This is especially interesting given the skeletal muscle biopsy performed at 3 months of age was abnormal, as detailed in the histology section below. 

Since the original report, two additional children were born to the same parents (as presented on the pedigree in Figure 1), including one unaffected and one affected male, presented below. The parents report no abnormal cardiac symptoms such as shortness of breath or chest pain but have not had formal cardiac evaluations despite recommendations to follow up with cardiology and have echocardiograms performed.

### 3.2. Clinical Description of the Male Proband (P2)

P2 was born by forceps-assisted vaginal delivery at 38 weeks and 5 days gestation to unaffected and healthy parents in a consanguineous relationship (first cousins). This child was the seventh pregnancy for these parents. After the birth of P1 (the parent’s 4th pregnancy), the parents experienced one healthy pregnancy that resulted in a healthy male child, one early first trimester miscarriage and, finally, the birth of P2, followed by one early termination of pregnancy due to positive prenatal testing for the homozygous *MYBPC3* c.1224-52G>A (IVS13-52G>A) variant, as presented in Figure 1 and Table 1.

In utero imaging of P2 was suspicious for left ventricular cardiomyopathy and a moderate apical ventricular septal defect. One day prior to delivery, an ultrasound noted oligohydramnios, appropriate fetal growth and normal doppler studies. A postnatal echocardiogram confirmed a mildly dilated left ventricle with prominent trabeculations, mildly decreased left ventricular function (LVEF 48%), mild-to-moderately decreased right ventricular systolic function and a small apical muscular ventricular septal defect. Repeat echo at one week of life revealed the progression of LV dysfunction with an LVEF of 12%. He was stabilized with milrinone and furosemide infusions, and mechanical ventilation. At 5 weeks of age, his LVEF was stable at 44% on a milrinone infusion and no respiratory support. He was weaned off milrinone at 7 weeks of life to oral heart failure therapies. 

Clinically, P2 was stable with outpatient medical management of his cardiac dysfunction until the age of 4 months, at which point he exhibited lethargy, tachypnea, irritability, poor oral intake and weight loss. At that time, he was admitted to the cardiac intensive care unit for acute on top of chronic heart failure. After 2 weeks of aggressive medical management and failure to improve symptoms, he underwent placement of a left ventricular assisted device and closure of the ventricular septal defect (VSD) and patent foramen ovale (PFO). He remained admitted to the hospital for roughly 5 months until a successful heart transplantation at 9 months of age was performed. His postoperative course was uneventful. In terms of his development, P2 is currently 19 months old and is developmentally doing well and meeting milestones. He initially had mild residual delays secondary to his prolonged hospital course and mechanical circulatory support but is currently thriving at home with his family. 

### 3.3. Identification of SMYD1 and MYBPC3 Variants in New Patients

Whole exome sequencing (WES) performed on DNA from the family revealed that P1 is homozygous for a novel variant c.302A>G (p.Asn101Ser) in the *SMYD1* gene (GenBank: NM_198274.3), as well as homozygous for a variant c.1224-52G>A (IVS13-52G>A) in the *MYBPC3* gene (GenBank: NM_000256.3). Her sibling, P2, is homozygous for the same *MYBPC3* variant c.1224-52G>A (IVS13-52G>A). A summary of the variants identified from WES or targeted testing is presented in Table 2. Both parents are heterozygous for both *SMYD1* and *MYBPC3* variants, and the two living siblings are unaffected and do not carry the familial *SMYD1* and *MYBPC3* variants (Figure 1 and Table 1). 

**Figure 2:**
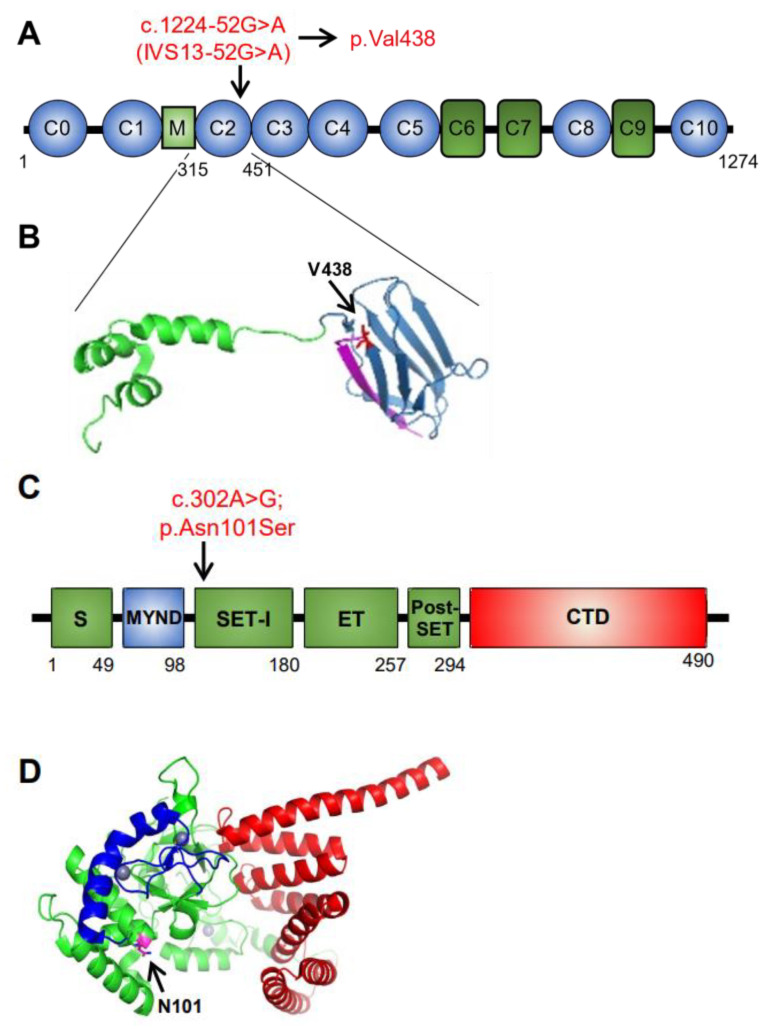
Identification of a *MYBPC3* (c.1224-52 G>A (IVS 13052 G>A) and novel *SMYD1* (c.302A>G; p.Asn101Ser) variant in humans. (**A**) Linear representation of the predicted structural domains of human MYBPC3 containing modules with immunoglobulin (blue ellipses) and fibronectin folds (green squares) termed C0 through C10 and an unstructured M domain (light green square) called the myosin S2-binding region. A truncating variant that was identified in both P1 and P2 and that leads to the premature stop codon at V438 is indicated with an arrow. (**B**) The NMR solution structure of the M domain trihelix bundle and C2 of human cardiac myosin-binding protein C (PDB ID: 5K6P) showing the amino acid Valine at position 438 (in red), where the premature stop codon is located. (**C**) Linear representation of the predicted structural domains of the SMYD1 protein in humans based on published mouse SMYD1 domains. A single amino acid substitution at position 101 (Asn to Ser) that was identified in P1 is indicated with an arrow. (**D**) The crystal structure of the entire mouse SMYD1 protein (PDB ID: 3N71) showing the location of residue 101 at the substrate binding site.

The specific *MYBPC3* c.1224-52G>A (IVS13-52G>A) variant generates a cryptic acceptor splice site within intron 13, causing 50 bp intronic sequence inclusion in the mRNA and expansion of exon 14. This new reading frame leads to a premature stop codon at amino acid position 438, which is 30 amino acids post-Gly407, the first amino acid of exon 14 [17] (Figure 2A,B). This *MYBPC3* variant has been previously identified and reported in adult patients in a few publications (as summarized in Table 3) and is classified as a “pathogenic or likely pathogenic” variant according to ClinVar with gnomAD MAF of 0.00003191 [37]. 

The novel *SMYD1* variant is a single nucleotide substitution at position 302 from adenine to guanine (c.302A>G), leading to an amino acid substitution at position 101 from asparagine to serine (abbreviated as Asn101Ser or N101S), as shown in Figure 2C,D. This *SMYD1* variant is currently classified as a “variant of uncertain significance (VUS)”, because it is a novel missense variant with no previous association to human disease or specific functional studies to this variant before this report, although there is strong evidence in animal models linking perturbations in *SMYD1* to cardiomyopathies. In Table 4, we present information about reported variants in the human *SMYD1* gene that were identified in patients with cardiac disease, supplemented with additional information on gnomAD constrain values and the prediction of in silico algorithms for comprehensiveness of the information regarding these variants.

### 3.4. Myocardial and Skeletal Biopsy Pathology

While MYBPC3 is a cardiac-specific isoform of the MYBPC protein, SMYD1 displays tissue-specific expression restricted to striated muscle that includes cardiac and skeletal tissue [28]. Since this unique pattern of expression, both cardiac and skeletal tissues that were collected from P1 were evaluated for histopathological changes. The cardiac tissue that was collected from P2 was subjected to histopathological analysis as well.

**Figure 3:**
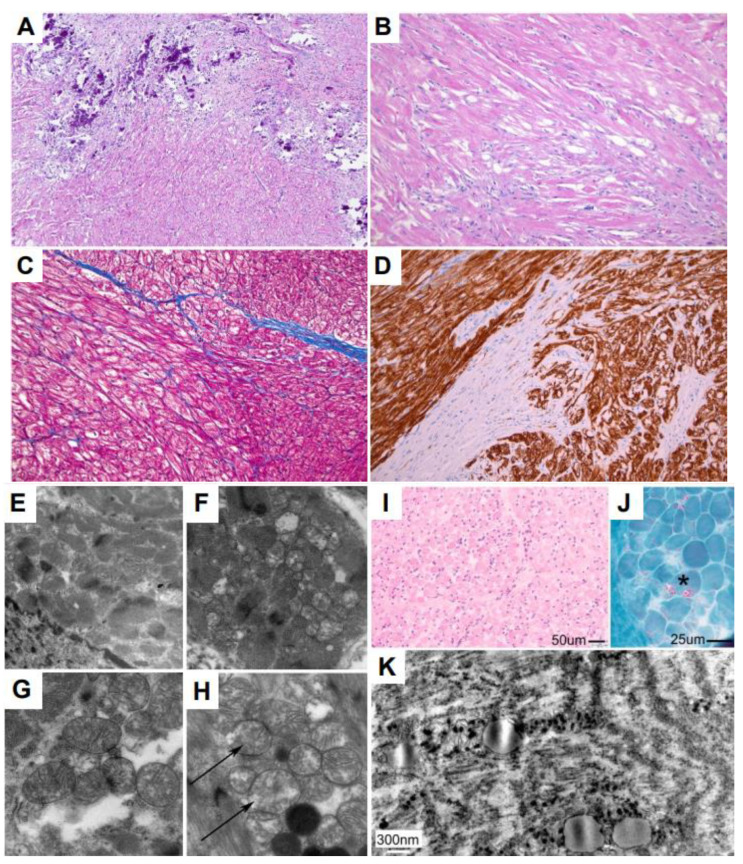
Histological and ultrastructural analysis of cardiac tissue and skeletal muscle from P1. Cardiac Tissue: (**A**) Myocardium photomicrograph showing multifocal calcification and areas of fibrosis replacing myocardial fibers (H&E; 100×). (**B**) Higher magnification showing a disarray of myocardial fibers (H&E 200×). (**C**) Collagen fibers appear in blue on this Masson’s trichrome stain between myocardial fibers (Masson’s trichrome; 200×). (**D**) Immunohistochemical staining for desmin, an intermediate filament characteristic of muscular tissue showing a loss of myocardial fibers replaced by fibrosis (IHC for desmin; 200×). (**E**,**F**) Myocardial fibers show the mildly altered architecture of the sarcomeric elements between which there are mitochondria with reduced and irregular cristae (magnifications: E = 8900×; F = 11,000×). (**G**) Several mitochondria show a loss of cristae (magnification: 18,000×). (**H**) The loss of mitochondrial cristae is extensive, with some mitochondria almost completely devoid of them (arrows; magnification: 22,000×). Skeletal Muscle: (**I**) Hematoxylin and eosin-stained frozen sections demonstrate a moderately increased variation in myofiber size with a few hypertrophic fibers and many scattered atrophic small fibers. There is no inflammation, and there are no definitive degenerating fibers. (**J**) Gomori’s trichrome stain identifies very rare fibers with vacuoles containing red granules (asterisk). (**K**) Ultrastructural studies show abnormal mitochondria with osmophilic inclusions and abnormal cristae.

Examination of the P1 cardiac tissue sections showed markedly abnormal myocardium with extensive fibrosis and multifocal calcifications. The remaining cardiomyocytes exhibited variable sizes, with an irregular, haphazard architecture and areas of cytoplasmic vacuolization. No acute or chronic inflammation was identified within the myocardium (Figure 3A–F). Additional high-power ultrastructural images show the presence of abnormal mitochondria with reduced and irregular cristae or loss of cristae ranging from mild to extensive loss (Figure 3E–H).

Muscle biopsy of the right thigh from P1 showed atrophy affecting both type 1 and 2 fibers without inflammation or myofiber necrosis (Figure 3I). There was mild myofibrillar disorganization seen on NADH-tetrazolium reductase reacted sections (not shown). Rare vacuoles were identified containing eosinophilic material on Gomori’s trichrome stain (Figure 3J). No ragged red fibers were present. Ultrastructural studies confirmed myofibrillar disorganization and demonstrated an overall paucity of mitochondria. High-power ultrastructural images identified abnormal mitochondria with osmophilic irregular or rectangular inclusions and abnormal cristae (Figure 3K).

**Figure 4:**
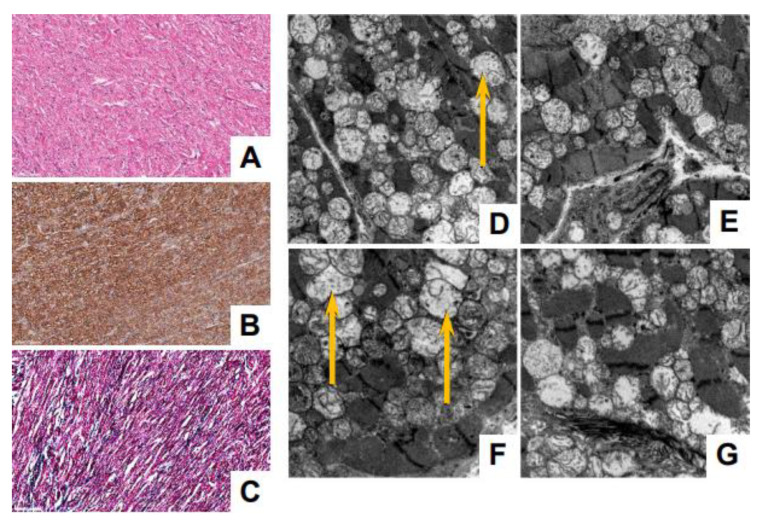
Histological and ultrastructural analyses of cardiac tissue from P2. (**A**–**C**) Light microscopy evaluation indicates a preserved architectural myocardial pattern, free of fibrosis and calcifications. (**B**) Immunohistochemical staining with desmin, showing normal preservation of this intermediate muscle-specific filament. Scale bar = 100 µm. (**D**–**G**) Ultrastructural analysis indicates myocardial fibers that show the preserved architecture of the sarcomeric elements; the mitochondria demonstrate reduced and irregular cristae (magnifications: E = 4800×; FG = 6800×). (**D**,**F**) Extensive mitochondrial loss of cristae, with some mitochondria almost completely devoid of them, as indicated by arrows (magnification: D = 4800×).

The histopathological analysis of cardiac tissue from P2 did not reveal significant changes in the morphologic appearance of the myocardium at the light microscopy level (Figure 4A–C). The ultrastructural analysis demonstrates the preserved morphology of the sarcomeres but profoundly abnormal mitochondria with extensive loss of cristae (Figure 4D–G).

## 4. Discussion

Infantile-onset cardiomyopathy is a rare disease, and when diagnosed, it carries a substantial risk of morbidity and mortality [2,38,39]. Genetic testing is recommended for cardiomyopathies per the guidelines of the American College of Medical Genetics and Genomics (ACMG) [40]. While there are several types of cardiomyopathies with significant genetic heterogeneity and overlap in the phenotype [41,42], sarcomeric gene mutations associated with cardiomyopathy are the most common and have been found in patients presenting from infancy to adulthood [3,40,43].

The *MYBPC3* gene encodes the cardiac isoform of myosin-binding protein C, a phosphoprotein located in the central region of the A-band of the sarcomere that plays an important role in the regulation of cardiac contractility. Its phosphorylation is essential for myosin–actin interaction and, therefore, for force production. Through its interaction with myosin, titin and actin, it contributes to the structural integrity of the sarcomere, playing an important role in thick filament assembly and stability. Pathogenic variants in *MYBPC3* are one of the main causes of hypertrophic cardiomyopathy (HCM) development. Previous reports have described many variants identified in the *MYBPC3* gene [44,45]. Around 75% of these variants were found to be loss-of-function and resulted in absent or truncated proteins with impaired ability to bind to other sarcomeric proteins, being the main reason for the development of hypertrophic cardiomyopathy in both pediatric and adult populations [44,45]. To date, this specific *MYBPC3* c.1224-52 G>A (IVS13-52 G>A) variant has only been identified in adult patients with hypertrophic cardiomyopathy containing a heterozygous variant or unknown heterozygosity [17,18,19,20,21] (summarized in Table 3). This specific truncation occurs in the immunoglobulin-type C2 domain, which forms a compact globular structure. It has been proposed that domains C2, C3 and C4 are required to provide the N-terminal region with sufficient flexibility so that the interaction occurs with the S2 region of myosin or with the actin filament [9,46]. The presence of this variant is expected to produce an abnormal transcript that could be rapidly degraded before translation by the nonsense-mediated mRNA decay pathway. 

The vast majority of the *MYBPC3* variants that lead to hypertrophic cardiomyopathy are present in adult populations and have been associated with late-onset disease [12,47]. To date, there is very limited data available to link *MYBPC3* variants to pediatric onset HCM. This includes a common founder Amish mutation in the *MYBPC3* gene producing the splice variant c.3330+2T>G. Individuals that are heterozygous for this variant manifest with HCM in adulthood, while individuals that are homozygous for this variant present with severe infantile HCM that could be lethal unless they receive a heart transplant [48,49]. There are no reports describing a specific phenotype of the homozygous *MYBPC3* c.1224-52 G>A (IVS13-52 G>A) variant in pediatric patients with cardiac disease. Here, we report the clinical presentation of two infants carrying a homozygous variant c.1224-52 G>A (IVS13-52G>A) in the *MYBPC3* gene and presenting with severe infantile cardiomyopathy. P2 (homozygous for this variant) presented with progressive biventricular dysfunction in the first week of life. He responded to aggressive medical therapy and remained clinically stable for the first 4 months of life before deteriorating to the point of needing mechanical circulatory support. Interestingly, the histopathological evaluation of the cardiac tissue from P2 did not reveal significant changes in the myocardium morphology, including sarcomere integrity. On the other hand, the histopathological analysis from cardiac tissue from P1 indicated markedly abnormal myocardium with extensive fibrosis and multifocal calcification. P1, who is homozygous for both the *MYBPC3* c.1224-52 G>A (IVS13-52 G>A) and *SMYD1* c.302A>G (p.Asn101Ser) variants, presented in a similar fashion to P2 but progressed more rapidly to mechanical circulatory support. P1 began experiencing cardiac complications three days after birth and declined rapidly over the next 2 months, at which time the patient received a BiVAD as a bridge to cardiac transplantation [36]. The additional histological analysis of skeletal muscle from P1 highlights abnormalities in myofibrillar organization in both cardiac and skeletal muscle that is consistent with observations in zebrafish, which showed that SMYD1 was necessary for sarcomere assembly and organization [50,51,52]. It is intriguing that P1 retained the ability to walk with no signs of hypotonia at the age of 2 years and 11 months during the clinical post-op evaluation and, most recently, at almost 6 years of age, even with the notable histological changes in the skeletal muscle. This could be due to the fact that it is too early to see clinical signs of skeletal muscle involvement during childhood. In addition, the ultrastructural analysis of cardiac tissue from both P1 and P2 revealed profoundly abnormal mitochondria with grossly reduced or lost cristae. Currently, there are very limited studies linking hypertrophic cardiomyopathy with pathogenic variants in *MYBPC3* to mitochondrial abnormalities and mitochondrial dysfunction [53], and this specific variant c.1224-52 G>A, to our knowledge, was not linked previously to mitochondrial abnormalities and/or dysfunction. Thus, future studies will be necessary to identify the specific molecular and pathophysiological mechanisms leading to these mitochondrial abnormalities.

Although the initial WES analysis of P1 first identified this novel *SMYD1* c.302A>G (p.Asn101Ser) variant (as briefly mentioned in the case report published in 2019 [36]), the subsequent reanalysis of data from this patient revealed the presence of a second pathogenic variant in *MYBPC3* c.1224-52 G>A (IVS13-52G>A). The more rapid progression to infantile cardiomyopathy in the P1 patient may suggest an additive effect of both variants, since P1 had a more rapid progression and need for mechanical circulatory support by 2 months of age, as compared to P2, who is homozygous for the *MYBPC3* variant only. However, it is likely the cardiac dysfunction may be solely or largely driven by the *MYBPC3* variant, with minimal or no contribution from the *SMYD1* c.302A>G (p.Asn101Ser) variant. Nevertheless, it is tempting to speculate that the *SMYD1* c.302A>G (p.Asn101Ser) variant may contribute to the cardiac dysfunction in P1, considering the fact that *SMYD1* is expressed in both heart and skeletal muscle. *MYBPC3* is expressed exclusively in heart muscle [4], not in skeletal muscle, where we also observe a similar cellular phenotype as cardiac tissue with abnormal mitochondrial morphology. Ultimately, additional functional studies will be necessary to determine the individual contribution of the *SMYD1* and *MYBPC3* variants to the phenotype, including in vitro and in vivo studies targeting each variant individually and in combination.

## Figures and Tables

**Table 1:** Whole exome sequencing/familial genetic testing results from the proband’s family showing variants in *MYBPC3* and *SMYD1*.

Relationship	Affected (Infantile Cardiomyopathy)	*MYBPC3* (c.1224-52G>A: IVS13-52G>A)	*SMYD1* (c.302A>G; p.Asn101Ser)	Current Age (Years)
Father	No	Heterozygous	Heterozygous	40
Mother	No	Heterozygous	Heterozygous	29
Male	No	No	No	7
Female (P1)	Yes	Homozygous	Homozygous	5
Male	No	No	No	4
Male (P2)	Yes	Homozygous	No	1
TOP	Unknown	Homozygous	Not done	Pregnancy terminated

Yellow highlights indicate the affected individuals (P1 and P2). TOP: termination of pregnancy.

**Table 2:** Gene variants identified by whole exome sequencing in P1.

Gene	Mode of Inheritance	Variant	Reference Sequence	Classification	Result
*SMYD1*	Unknown	c.302A>G, p.Asn101Ser	NM_198274.3	Variant of uncertain significance	Homozygous
*MYBPC3*	Autosomal Dominant/ Recessive	c.1224-52G>A, IVS13-52G>A	NM_000256.3	Pathogenic Variant	Homozygous

**Table 3:** Studies reporting the *MYBPC3* c.1224-52G>A variant in humans.

Reference	Phenotype	Zygosity	Number of Patients	Patient’s Age (Years)
Bagnall et al. [17]	Hypertrophic cardiomyopathy	unknown	3	23, 29, one unknown
Harper et al. [18]	Hypertrophic cardiomyopathy	heterozygous	OMGL * cohort: 32 of 2757 HCMR * cohort: 23 of 2636	OMGL cohort: 54.5 +/− 16.3 HCMR cohort: 49.5 +/− 11.3
Lopes et al. [20]	Hypertrophic cardiomyopathy	heterozygous	18	10–72
Holliday et al. [19]	Nonobstructive hypertrophic cardiomyopathy	heterozygous	1	48
Torrado et al. [21]	Hypertrophic cardiomyopathy	unknown	HIC * cohort: 12 of 9611	15–69, three unknown

* OMGL—Oxford Medical Genetics Laboratory; HCMR—Hypertrophic Cardiomyopathy Registry; HIC—Health In Code database.

**Table 4:** Reported variants in the human *SMYD1* gene identified in patients with cardiac diseases.

Nucleotide/Amino Acid Change	SMYD1 Protein Domain	Zygosity	Phenotype	gnomAD MAF *	PolyPhen-2	SIFT	Reference
c.814T>C p.Phe272Leu	Post-SET	heterozygous	Atrial enlargement, hypertrophic cardiomyopathy	0.000003979	Possibly damaging	Deleterious	[33]
c.302A>G p.Asn101Ser	SET-I	homozygous	Biventricular heart failure	0.00003589	Benign	Tolerated	[36], this report
c.675delA p.Lys225Asnfs*8	SET	heterozygous	Left ventricular noncompaction cardiomyopathy, arrhythmia	0.00009899	No PolyPhen-2 prediction available	No SIFT prediction available	[34]
c. 1321C>T p.Arg441Trp	CTD	heterozygous	Aortic valve stenosis and bicuspid aortic valve	0.006564	Possibly damaging	Deleterious	[35]

* MAF—minor allele frequency.

## Data Availability

Data is contained within the article.
